# Assessment of focused antenatal care utilization and associated factors in Western Oromia, Nekemte, Ethiopia

**DOI:** 10.1186/s13104-019-4311-3

**Published:** 2019-05-15

**Authors:** Ashenafi Habte Woyessa, Tahir Hasen Ahmed

**Affiliations:** 1 Department of Emergency and Critical Care Nursing, School of Nursing and Midwifery, Institute of Health Science, Wollega University, Nekemte, Ethiopia; 2 Department of Nursing, School of Nursing and Midwifery, Institute of Health Science, Wollega University, Nekemte, Ethiopia

**Keywords:** Focused, Antenatal care, Quality, Comprehensive, Western Ethiopia

## Abstract

**Objective:**

Despite the fact that quality antenatal care is one of the essential aspects in maternal and child health care, the current perceived quality and associated factors of this service is not well acknowledged in Ethiopia. This study was therefore undertaken to assess focused antenatal care service utilization and associated factors in western Ethiopia.

**Result:**

This study has measured the utilization of focused antenatal care services in terms of regularity of frequency of attendance, initiation time and completeness of the components. In about 19.8% of mothers attendance was irregular. While than three-fourths 330 (78.6%) started in the second trimester, and 42 (10%) of them commenced in the third trimester. The essential components of the services like counseling on nutrition, family planning, and HIV/AIDS were respectively missing in 1.9%, 8.3% and 7.4% of clients. Providing and receiving quality ANC was found to have emanated from different factors which were related to mothers, providers and facilities. Although the overall ANC utilization noticed deceivingly seems satisfactory, it was not fully comprehensive, focused and not to its current standard. Further efforts in terms of effective planning, monitoring and evaluation activities on the service are therefore strongly recommended.

## Introduction

It is palpable that more than half of maternal death occurs in sub-Saharan African. Most the deaths are preventable through provision of quality ANC. Antenatal care ensures healthy outcomes of women and newborns. It is also a key entry point for pregnant women to receive a broad range of health promotion and preventive health services. Moreover, it is one of the key factors in predicting the outcome of childbirth which helps detect early risk factors and potential complications of pregnancy [1].

Improving maternal and child health can only be realized through provisions of key services like quality and focused ANC. Not attendance alone but it is quality ANC service that would benefits the mothers and new born. Above all, receiving quality ANC services is a fundamental right for women to safeguard their health. In this regard, apart from reporting the overall coverage, how many of pregnant women receive standardized ANC services is obscured in Ethiopia [1, 2].

There is wide difference in its coverage between industrialized (98%) and low income countries (68%) countries. Although the coverage of ANC services is increasing in many African countries, coverage alone does not provide sufficient information on quality of the service. The traditional approach to ANC which is based on European models assumes that better care is achieved by frequent routine visits. However, evidence-based researchers have found that the practice of the traditional approach to ANC based on the European models to be wasteful and misleading. Moreover, this old approach is criticized to be less effective in terms of delivering quality ANC services [3, 4].

In many low-income countries, numerous obstacles do exist to the provision of focused and quality ANC services. Similar to other developing countries, unsatisfactory proportion of women are receiving antenatal care from a trained health professional during their pregnancy time in Ethiopia [4–8]. This is often related to an insufficient number of skilled providers particularly in rural and remote areas, socio- economic and cultural influences are among important barriers. A shortage in supplies can also be a problem in providing qualified ANC services [9–11].

Furthermore, traditional beliefs of the pregnant women and their awareness on care of pregnancy, knowledge of ANC services in terms of benefits, time to begin, and frequency to attend are additional factors related ANC utilization. More importantly, poor attitudes of service providers including lack of respect, privacy, confidentiality, providers at ANC clinics may negative influence on women´s use of ANC [11, 12].

Although ample studies have been done on ANC in Ethiopia, probably none of them has gone beyond reporting simply the coverage of the service. Information indicating the current status of focused antenatal care service including factors affecting quality of the service is critically limited in many parts of Ethiopia. The objective of this study was therefore to assess the focused antenatal care utilization and associated factors among pregnant women attending ANC in western Ethiopia. The findings of this study would also be instrumental for health planners and stakeholders working on maternal health.

## Main text

### Study area and period

This study was conducted in selected zonal hospitals (Nekemte, Gimbi and Shambu hospitals) located in west Oromia region.

### Study design and sampling techniques

A descriptive cross-sectional study design was used to conduct the study. Systematic sampling technique was used to select the study participants. Participants for in-depth interview were randomly selected from mothers who were attending their ANC for the fourth time and above. Accordingly, a total of 13 respondents were proportionally selected from each hospital. The sample was determined using single population proportional formula at the p-value of 50%, with the margin of error 5%, 95% confidence interval with considering 10% nonresponse rate making the total sample size of 422.

### Survey instrument and data collection process

Modified standardized questionnaire was used to collect the required data. The questionnaire was comprised of socio-demographic, reproductive and obstetric history, perceived quality of ANC and factors influencing the Utilization of ANC. The required data was collected by face- to face interview. To get deep insight of focused ANC and associated factors selected questions were provided for in-depth interview. The questions utilized for this purpose included and did not limit to adequacy of time providers take with mothers, decision making abilities during the service, the benefits of ANC, and possible barriers to quality ANC services and overall comfort on the service.

### Data collection, quality control, and analysis

Pretest was carried out to test the quality of the questionnaires. Each questionnaire was checked for completeness and consistency. Face of face interview was made by six data collectors who were not staff of the selected hospitals in order to avoid biases. Quantitative data was initially entered into Epi-data version 7. Then, it was exported to SPSS version 20 for data analysis. Qualitative data from in-depth interview was analysed narratively after they have been transcribed.

### Results

#### Socio-demographic characteristics of respondents

There were 420 respondents participated in this study. Nearly half (49%) of them were between of 18 and 25 years. One hundred ninety (45.2%) of the mothers were between 26 and 35 years. The mean ages of the participants were 26.3 with the standard deviation of 4.8. Regarding the maternal educational status, 87.9% of the respondents have attended formal education. The rest (12.1%) had no formal education.

#### Base line information of the mothers

Around 27% of mothers were married before the age 18 ears. While almost one-fourth (25.2%) of them had 1–2 pregnancies, 174 (41.4%) and 140 (33.3%) had respectively 3–4 and more than five pregnancies. With regard to the age at first delivery, 54 (12.9%) of mothers had delivered less than 18 years. Three-fourth 309 (73.6%) of mothers delivered between ages of 18–25 years. As to the number of abortions a woman faced, 28 (6.7%) and 3 (.7%) faced it once twice respectively. Majority (94.8%) of respondents reported that they did not encounter child deaths.

#### ANC utilization and associated factors

This study has measured utilization of ANC in terms of regularity, frequency of attendance, initiation time, and completeness of components of ANC. Attendance was irregular in 19.8% of mothers; Majority 330 (78.6%) of them started the service in the 2nd trimester. Few important components of the ANC were also missing (Table 1).

**Table 1:** Focused antenatal care utilization among pregnant women in west Oromia, Nekemte, Ethiopia, 2017

Variables (N = 420)	Response	Frequency	%
Frequency of ANC attendance	Once	64	15.2
	Twice	76	18.1
	Three times	124	29.5
	Four or more times	135	32.1
	Didn’t attended	21	5.0
Information not given at ANC clinics	Nutrition in pregnancy	8	1.9
	Care of the baby	11	2.6
	Family planning	35	8.3
	Place of delivery	12	2.9
	Progress of pregnancy	19	4.5
	Complications in pregnancy	18	4.3
	Counseling on HIV/AIDS	31	7.4
	None	286	68.1
Sites of ANC attendance	Private clinic	17	4.0
	Health centers	234	55.7
	Government hospital	151	36.0
	Missionary hospital	4	1.0
	Not attended	14	3.3
Trimester of ANC follow up	< 14 weeks	48	11.4
	14–27 weeks	330	78.6
	28–40 weeks	42	10.0
Regularity of ANC attendance	Yes	337	80.2
	Sometimes	69	16.4
	Never attended	14	3.3
Patterns of ANC attendance after booking	Appointment days	337	80.2
	When I have complaints	69	16.4
	I do not go at all	14	3.3
Attendance Time of ANC	In the morning	119	28.3
	In the afternoon	30	7.1
	Anytime I want	257	61.2
	Not at all	14	3.3

Providing and receiving quality ANC in this study was found to have emanated from different factors. Significant percentage (19.3%) the clients walked greater than 1 h. About 22.9% and 3.6% of mothers evaluated approach of providers as medium and not good respectively (Table 2). As overall, comparisons between selected variables were found to have statistical association with focused ANC utilization (Table 3).

**Table 2:** Factors influencing focused ANC utilization for mothers attending hospitals in western Oromia, Nekemte, Ethiopia, 2017

Variables (N = 420)	Responses	Frequency	%
Proximity of health institutions	< 15 min	60	14.3
	15–30 min	181	43.1
	30–60 min	98	23.3
	> 60 min	81	19.3
Sources of ANC information	Health institutions	75	17.9
	Radio\TV	32	7.6
	TBA	5	1.2
	Health extension workers	230	54.8
	Friends and relatives	72	17.1
	Don’t remember	6	1.4
Approaches of the health care providers	Good	309	73.6
	Medium	96	22.9
	Not good	15	3.6
Language barrier	Yes	61	14.5
	No	359	85.5
Cultural acceptance	Yes	363	86.4
	No	57	13.6
Religious acceptance of the services rendered	Yes	375	89.3
	No	45	10.7
Husband’s acceptance of the services rendered	Yes	387	92.1
	No	33	7.9
Mothers involvement in ANC related decision making	Fully	190	45.2
	Partially	189	45.0
	Not involved at all	41	9.8
Mothers waiting time in health institution	10–30 min	181	43.1
	30–60 min	177	42.1
	> 60 min	62	14.8
Awareness of mothers reasons of ANC check up	Maternal health	14	3.3
	Child health	11	2.6
	Both	378	90.0
	Do not know	17	4.0

**Table 3:** Comparison of selected variables with focused ANC utilization for mothers attending hospitals in western Oromia, Nekemte, Ethiopia, 2017

Variables	Category	Regular ANC attendance		OR (95% CI)	P-value	AOR (95% CI)	P-value
		Yes	No				
Age of mother	< 18	8 (2.0%	0 (.00)	.000	.042	.000	.612
	18–25	169 (49.8%)	37 (28.6%)	.219 (.077, .621)	.999	.474 (.121, 1.849)	.999
	26–35	152 (45.6%)	38 (35.7%)	.250 (.088, .709)	.004	.415 (.113, 1.526)	.282
	>35	8 (2.7%)	8 (35.7%)	1 (R)	1 (R)	1 (R)	1 (R)
Maternal educational	No formal education	32 (9.5%)	19 (22.9%)	3.592 (1.716, 7.521	.001	1.198 (.407, 3.530)	.809
	Grade 1–8	106 (31.5%)	25 (30.1%)	1.427 (.750,2.715)	.279	.799 (.336, 1.901)	.743
	Grade 9–12	78 (23.1%)	19 (22.9%)	1.474 (.740, 2.936)	.270	1.009 (.458, 2.223)	.611
	College level (+)	121 (35.9%)	20 (24.1%)	1 (R)	1 (R)	1 (R)	1 (R)
Residence	Rural	91 (22.4%)	6 (42.9%)	1.839 (1.083,3.122)	024	1.388 (.446, 1.772)	.738
	Urban	315 (77.6%)	8 (57.1%)	1 (R)	1 (R)	1 (R)	1 (R)
Paternal educational status	No formal education	15 (4.7%)	8 (28.6%)	3.002 (1.184,7.611)	.021	1.653 (.391, 4.926)	.612
	Grade 1–8	44 (14.5%)	19 (28.6%)	2.431 (1.273, 4.642)	.007	1.311 (.666,4.101)	.278
	Grade 9–12	81 (24.4%)	21 (21.4%)	1.459 (.801, 2.658)	.217	.627 (.596,2.883)	.501
	College level (+)	197 (56.4%)	35 (21.4%)	1 (R)	1 (R)	1 (R)	1 (R)
Maternal own income	Yes	179 (53.1%)	32 (38.6%)	.554 (.339, .905)	.018	.275 (.342,1.148)	.099
	No	15 (46.9%)	51 (61.4%)	1 (R)	1 (R)	1 (R)	1 (R)
Total family size	< 3	158 (44.8%)	29 (35.7%)	.245 (.052, 1.151)	.075	.677 (.036, 2.125)	.228
	4–6	146 (43.1%)	32 (21.4%)	.292 (.062, 1.370)	.119	1.377 (.446, 1.662)	.716
	7–10	29 (10.8%)	19 (28.6%)	.874 (.176,) 4.348	.869	.199 (.038, 2.185)	.351
	> 10	4 (1.2%)	3 (14.3%)	1 (R)	1 (R)	1 (R)	1 (R)
Number of under 5 children	None	173 (48.8%)	31 (42.9%)	.090 (.008, 1.018)	.052	.208 (.083, 5.538)	.431
	One	146 (45.6%)	43 (28.6%)	.147 (.013, 1.663)	.121	3.766 (.459,2.828)	.304
	Two	17 (5.4%)	7 (14.3%)	.206 (.016, 2.655)	.226	3.131 (.011, 3.583)	.043^a^
	Three	1 (.2%)	2 (14.3%)	1 (R)	1 (R)	1 (R)	1 (R)
How many times she faced abortion	None	318 (94.4%)	71 (85.5%)	2.898 (1.301, 6.455)	.009	.753 (.018,, 5.526)	.841
	Once	17 (5.0%)	11 (13.3%)	2.239 (.200, 25.039)	.513	.906 (.013,21.401)	.746
	Twice	2 (.6%)	1 (1.2%)	1 (R)	1 (R)	1 (R)	1 (R)
Involved in decision making	Fully	157 (46.6%)	33 (39.8%)	1 (R)	1 (R)	1 (R)	1 (R)
	Partially	153 (45.4%)	36 (43.4%)	1.119 (.664, 1.887)	.672	.644 (.498, 1.647)	.232
	Not at all	27 (8.0%)	14 (16.9%)	2.467 (1.169, 5.205)	.018	3.766 (.459,2.929)	.397
Accessibility to ANC services	Yes	303 (89.2%)	66 (10.8%)	.436 (.230, .826)	.011	.474 (.742, 3.425)	.013^a^
	No	117 (20.5%)	354 (79.5%)	1 (R)	1 (R)	1 (R)	1 (R)
Cultural acceptance	Yes	297 (88.1%)	66 (79.5%)	1.912 (1.022, 3.580)	.043	.415 (.232, 1.783)	.999
	No	40 (11.9%)	17 (20.5%)	1 (R)	1 (R)	1 (R)	1 (R)
Religious acceptance	Yes	309 (91.7%)	66 (79.5%)	2.843 (1.471,5.492)	.002	1.198 (1.323,10.7)	.186
	No	28 (8.3%)	17 (20.5%)	1 (R)	1 (R)	1 (R)	1 (R)

^a^Statistically significant at P < .05

#### Result of qualitative part on focused antenatal care utilization

Regarding the adequacy of time providers take with mothers during ANC visits, one respondent replied, “*they gave us sufficient time with no any problems*”. The second respondent replied, *“There were many problems in the card room. After we were referred from the card room to this ANC clinic due to the large number of pregnant mothers who were waiting, they told us to go away and wait for their turns. Some mothers can wait from morning to the evening and can go home without getting the service and return another time. It is not to get sufficient time to discuss with HCW but entering to the ANC room is difficult. They didn’t give us sufficient time to discuss our problems with them*.” The third respondent said, “*My ANC appointment date is every 24th day. Last month the health care workers told me to return back the next day and when I came on my appointment day, again she told me to return back to the third time today i.e. on 26. I have discontinued my drugs (Iron and folic acid tabs)*”.

On the mothers’ decision making abilities during the service, another respondent complained by stating, “*We didn’t have any decision making ability simply they examined us and told us something to do and send us to our home. We only receive what they told us but not what we tell them*”. For a question on the benefits of ANC, two mothers answered, “*It helped me to know my own and my fetal health status” another mother said, “They weigh us; they measure the fetal growth*”.

In relation to the time of first visits, two mothers reported that they went at 2nd month of amenorrhea. Three mothers respectively started ANC at the 3rd, 4th, and 5th months of their pregnancy. On the overall comfort with ANC services, one respondent replied, “*We were examined in harsh way, so we cannot say it is good or bad”.* All the respondents had confirmed that there was no cultural and religious opposition against utilization of ANC. All of the mothers also mentioned that their husbands were cooperative and supported them in attending ANC.

### Discussion

This research has assessed the focused ANC utilization and associated factors in Western Oromia. Key findings related to base line information of respondents, and ANC utilization and associated factors are compared and contrasted in the context of other similar studies and briefly presented.

In this study, 95% of the mothers attended ANC at least once. This is much higher than the recent report in Ethiopia which showed only 34% of women receive the service at least. But it is still lower than the report from industrialized countries and this finding is nearly similar with study undertaken in Tanzania (94%) and higher than that of Malawi (49%) [3, 5, 13–15].

Quality of ANC services can be assessed by looking at the type of providers, the number of visits and the timing of the first visit [12, 13]. The findings of this study showed that 337 (80.2%) of the respondents attended ANC follow up on their appointment days. This finding is higher than the findings in the rest of developing countries (65%) but lower than that of developed countries which is 97% [16]. According to the findings of this study, more than three-fourths 330 (78.6%) of clients have initiated the services late in the second trimester (14–27 weeks). This finding is consistent with the study in Niger Delta and Kenya and better than the 48% of Malawi study [17, 18].

Generally, only 135 (32.1%) pregnant women were found to attain the WHOs’ recommendation of four ANC visits during pregnancy. This in line with findings many studies in African and it is even higher than a cross-sectional study done in Nigeria which found 20.3% [11, 19].

Ideally, one measures of quality of ANC is comprehensiveness of the service. In present study we identified that the rendered service was not as comprehensive as required. Essential aspects of ANC service like counseling on nutrition during pregnancy, teaching mothers about care of the baby and counseling on family planning were respectively in 1.9%, 2.6%, and 8.3% of respondents.

Providing and receiving quality ANC is related to many challenges. This encompasses lack of qualified providers, socio-economic and cultural beliefs are among important barriers identified in many similar studies conducted across the world [9, 12, 20]. Current study has identified factors influencing ANC utilization that were found to have related to mothers, providers and facilities. In general, comparisons between selected variables showed that ANC utilization was affected by a number of factors which were also statistically proven to be significant. Finally, response from in in-depth interview reflected that the ANC services being rendered were not as comprehensive and as qualified as required. Some of the participants have responded unfavorable answers for the raised questions in the in-depth interview. These findings are uniformly consistent with studies undertaken in similar context of elsewhere [21–26].

As overall, the findings of this study imply that although the overall ANC utilization deceivingly seems satisfactory, it was not as quality and not focused as expected. Many factors were identified to have contributed for the noticed unsatisfactory utilization of focused ANC indicating that many efforts are still needed.

## Limitations of the study

As the design of this research was institution based, it did not know some other possible barriers of the service among mothers who did not visit ANC clinics.

## Data Availability

The raw data supporting our findings are available from authors on a reasonable request.

## Abbreviations

ANC: Antenatal care
CHW: Community Health Workers
DHS: Demographic Health Survey
FANC: focused antenatal care
MDG: millennium development goal
MMR: maternal mortality rate
MNH: Maternal and Neonatal Health
TDHS: Tanzania Demographic and Health Survey
WHO: World Health Organization

## References

[1] Abou-Zahr CL, Wardlaw TM (2003). Antenatal care in developing countries: promises, achievements and missed opportunities: an analysis of trends, levels and differentials, 1990–2001.

[2] USAID (2007). Focused Antenatal care: providing integrated, individualized care during pregnancy.

[3] UNICEF, WHO. Data from Demographic and Health Surveys (DHS), Multiple Indicator Cluster Surveys (MICS) and other national surveys, late 1990s to 2001. 104 countries. Averages weighted by number of births 2002.

[4] Anh TT. Factors related to the acceptance of the new antenatal care protocol among health personnel in SuphanBuri Province, Thailand. [M.P.H.M Thesis in Public Health]. Bangkok: Faculty of Graduate Studies, Mahidol University; 2002.

[5] Centeral Statistics Agency, ICF Macro Calverton (2011). Ethiopia demographic and health survey 2011.

[6] Ornella L, Seipati M-A, Patricia G, Stephen M. Antenatal Care. Opportunity for African’s newborn, 2003.

[7] Yared M, Mekonnen A (2002). Utilization of maternal health care services in Ethiopia.

[8] Hunt P, Bueno De Mesquita B (2000). Reducing maternal mortality: the contribution of the right to the highest attainable standard of health.

[9] Asundep NN, Carson AP, Turpin CA, Tameru B, Agidi AT, Zhang K, Jolly PE (2013). Determinants of access to antenatal care and birth outcomes in Kumasi, Ghana. J Epidemiol Glob Health.

[10] Lincetto O, Mothebesoane-Anoh S, Gomez P, Munjanja S. Antenatal care. Opportunities for Africa’s Newborns: Practical data, policy and programmatic support for newborn care in Africa, Cape Town, South Africa: WHO, 2006, 51–62.

[11] Agus Y, Horiuchi S, Porter SE (2012). Rural Indonesia women’s traditional beliefs about antenatal care. BMC Res Notes.

[12] Dennis LI, Flynn BC, Martin JB (1995). Characteristics of pregnant women, utilization, and satisfaction with prenatal services in St. Petersburg, Russia. Public Health Nurs.

[13] Ministry of Health and Population (MOHP) [Nepal], New ERA, Kathmandu, Nepal and Macro International Inc., Calverton, Maryland, USA. Demographic and Health Survey, Nepal; 2007. p. 135–55.

[14] ZDHS. The Zambia Demographic and Health survey key findings, 2007.

[15] Mrisho M, Obrist B, Schellenberg JA, Haws RA, Mushi AK, Mshinda H, Tanner M, Schellenberg D (2009). The use of antenatal and postnatal care: perspectives and experiences of women and health care providers in rural southern Tanzania. BMC Pregnancy Childbirth.

[16] Ndidi EP, Oseremen IG (2010). Reasons given by pregnant women for late initiation of antenatal care in the Niger Delta, Nigeria. Ghana Med J.

[17] Magadi MA, Madise NJ, Rodrigues RN (2000). Frequency and timing of antenatal care in Kenya: explaining the variations between women of different communities. Soc Sci Med.

[18] Malawi Demographic and Health Survey 2010. National Statistical Office (NSO), ICF Macro, National Statistical Office (NSO):ICF Macro: Zomba, Malawi and Calverton, Maryland USA: NSO and ICF Macro; 2011

[19] Aniebue UU, Aniebue PN (2011). Women’s perception as a barrier to focused antenatal care in Nigeria: the issue of fewer antenatal visits. Health Policy Plan.

[20] Orvos H, Hoffman I, Frank I, Katona M, Pal A, Kovacs L (2002). The perinatal outcome of pregnancy without prenatal care—a retrospective study in Szeged, Hungary. Eur J Obstet Gynecol Reprod Biol.

[21] World Health Organization Maternal mortality fact sheet No. 348. 2010; http://www.who.int/mediacentre/factsheets/fs348/en/index.html. Accessed 13 Dec 2011.

[22] Lesser J, Anderson NL, Koniak-Griffin D (1998). Sometimes you don’t feel ready to be an adult or a mom. The experience of adolescent pregnancy. J Child Adolesc Psychiatr Ment Health Nurs.

[23] Lee SH, Grubbs LM (1995). Pregnant teenagers’ reasons for seeking or delaying prenatal care. Clin Nurs Res.

[24] O’Callaghan MF, Bororkowski JG, Whitman TL, Maxwell SE, Keogh D (1999). A model of adolescent parenting: the role of cognitive readiness to parent. J Res Adolesc.

[25] Stoltzfus RJ, Dreyfuss ML, Chwaya HM, Albonico M (1997). Hookworm control as a strategy to prevent iron deficiency. Nutr Rev.

[26] Raatikainen K, Heiskanen N, Heinonen S (2007). Under-attending free antenatal care is associated with adverse pregnancy outcomes. BMC Public Health J.

